# Optimization of Process Parameters for Fabricating Polylactic Acid Filaments Using Design of Experiments Approach

**DOI:** 10.3390/polym13081222

**Published:** 2021-04-09

**Authors:** Chil-Chyuan Kuo, Jia-You Chen, Yuan-Hao Chang

**Affiliations:** 1 Department of Mechanical Engineering, Ming Chi University of Technology, No. 84, Gungjuan Road, New Taipei City 243, Taiwan; U07117134@mail.mcut.edu.tw (J.-Y.C.); U07117159@mail.mcut.edu.tw (Y.-H.C.); 2 Research Center for Intelligent Medical Devices, Ming Chi University of Technology, No. 84, Gungjuan Road, New Taipei City 243, Taiwan

**Keywords:** fused deposition modeling, polylactic acid, filament, design of experiments approach

## Abstract

The amount of wasted polylactic acid (PLA) is increasing because 3D printing services are an increasingly popular offering in many fields. The PLA is widely employed in the fused deposition modeling (FDM) since it is an environmentally friendly polymer. However, failed prototypes or physical models can generate substantial waste. In this study, the feasibility of recycling PLA waste plastic and re-extruded it into new PLA filaments was investigated. An automatic PLA filament extruder was first developed for fabricating new PLA filaments. This paper also discusses the process, challenges, and benefits of recycling PLA waste plastic in an effort to fabricate new PLA filaments more sustainable. It was found that it was possible to fabricate PLA filament using recycled PLA waste plastic. The production cost is only 60% of the commercially available PLA filament. The tensile strength of the developed PLA filament is approximately 1.1 times that of the commercially available PLA filament. The design of experiments approach was employed to investigate the optimal process parameters for fabricating PLA filaments. The most important control factor affecting the diameter of PLA filament is the barrel temperature, followed by recycled material addition ratio, extrusion speed, and cooling distance. The optimal process parameters for fabricating PLA filament with a diameter of 1.7 mm include the barrel temperature of 184 °C, extrusion speed of 490 mm/min, cooling distance of 57.5 mm, and recycled material addition ratio of 40%.

## 1. Introduction

Fused deposition modeling (FDM) [1,2,3,4] is a process of fabricating physical models using materials such as polylactic acid (PLA) [5,6,7,8,9,10], polyamide (PA), polycarbonate (PC), acrylonitrile butadiene styrene (ABS), investment casting wax, and medical grade ABS. The fabricated physical models were widely used for engineering applications, such as automotive, aerospace, and medical devices. In recent years, some topics about FDM technology were studied intensively by many researchers all over the world. Mohamed et al. [11] reviewed the research carried out so far in determining and optimizing the process parameters of the FDM process and described the trends in future FDM research. Several statistical designs of experiments were used for the determination of optimum process parameters. Lee et al. [12] developed a hybrid additive manufacturing (AM) system integrating low-cost fused deposition modeling machine and five-axis machining. It was found that the embedded material can reduce the warpage caused by the shrinkage during the FDM process. Drummer et al. [13] investigated the shrinkage of the PLA and tricalcium phosphate (TCP) built with FDM machine. The components of PLA/TCP with sufficient mechanical properties have potential application as scaffolds. Perez et al. [14] employed preheated platforms and temperature-controlled build envelopes to decrease part warpage during the FDM process. It was found that the acrylonitrile butadiene styrene reinforced with 5 wt.% TiO_2_ exhibited the highest ultimate tensile strength. Bernal et al. [15] proposed a methodology using the impulse excitation technique to obtain an effective isotropic Young’s modulus of FDM printed thermoplastic materials to be used in topology optimization design. It was found that the greatest variation of Young’s modulus between directions is less than 2.6%. In addition, the greatest relative error of the measured frequencies with respect to the desired frequencies in the topology optimization problem is less than 2.9%. Lin et al. [16] proposed a simple yet versatile algorithm to produce isotropic products via optimizing the printing path. The workpiece was first separated into distinct areas in terms of the printing sequence, which increases the efficiency of the fabrication process. The results of this study provide a significant role in printing molds that require isotropic properties.

Zekavat et al. [17] investigated the effects of fabrication temperature on mechanical properties of FDM parts using X-ray computed tomography. The specimens fabricated at lower temperatures have relatively lower tensile strength despite their considerably higher strain at break. It was found that the X-ray computed tomography provides great potential as a non-destructive tool for investigation and development of FDM process. Baca et al. [18] focused on the filament extrusion method using two modes. It was found that the multi-nozzle demonstrated a better performance in the build time. Conversely, the single nozzle displayed greater consistency in generating better quality materials. Paggi et al. [19] evaluated corn starch/cellulose acetate (SCA) specimens 3D-printed by the FDM method. It was found that high values of the flexural modulus for the specimens printed at 240 °C and 80% flow rate, and the fatigue tests demonstrated that the 230–90 SCA specimens were resistant to successive cyclic loads. Camposeco-Negrete [20] presented an experimental study centered to optimize five responses associated with FDM. It was found that the proposed method allowed for simultaneous optimization of all the observed variables for the 3D printing process. Liu et al. [21] developed a novel rectangular-circular grid filling pattern of FDM in cellular lattice structures. It was found that the filling mode and corresponding parameter settings can reduce material consumption and improve mechanical performance.

The design of experiment (DOE) is a structured method for conducting experiments. Zaman et al. [22] optimized FDM process parameters using design of experiment (DOE). It was found that the optimal combination include a layer thickness of 0.2 mm, number of layers on the outside of the part as 4, infill pattern of diamond, and an infill percentage of 70% as per the analysis of variance (ANOVA) and S/N ratio. Adnan et al. [23] used the DOE to investigate the springback behavior of AA6061 strip with non-uniform thickness. It was found that the thickness is the most significant parameter to formability. Zhou et al. [24] carried out the DOEs method to investigate the effects of a special nozzle structure on its outlet velocity uniformity. It was found that the thickness, length, inlet velocity, width, and water-cement ratio are the influential factors on the outlet velocity uniformity. In addition, a higher water-cement ratio and a lower inlet velocity should be chosen as long as it meets the requirements of plastering quality and efficiency. Azadeh et al. [25] carried out the DOEs method to select the optimum maintenance policy. It was found that the best scenario is associated with first-in, first-out policy for all six machines, which was consequently determined as the optimal maintenance policy. Effertz et al. [26] carried out the DOEs method to optimize the process parameters for friction spot welded aluminum alloy. It was found that the plunge depth is the leading parameter for obtaining the best lap shear force. Feng et al. [27] designed an automated two-staged multi-objective optimization tool for plastic injection molding and applied to industrial product using the DOEs method. It was found that the number of process parameters was compressed from 8 to 5 considering factors’ contribution percentage for reduction in time and computation cost. Cherief et al. [28] used the DOEs method to determine the effect of each factor on the ultimate compression stress, deformation, and absorption energy. It was found that the magnitude of compressive stress varied into a range of 25.32 to 36.38 MPa. In addition, the thickness has a significant effect on compression properties and failure behavior. Oemar and Chang [29] used the DOEs method to gain 9 combinations of experimental design from three processing parameters, namely chemical reagent concentration, activation temperature, and activation time. It was found that the optimal product yield and amorphous percentage are obtained when chemical reagent concentration, activation temperature, and activation time are chosen as 25 wt.%-600 °C-.0 h and 25 wt.%-600 °C-1 h, respectively. Abdulkadir et al. [30] discussed the process parameter selection through variation and application of acoustic emission monitoring technique for obtaining optimal surface roughness in ultra-high precision turning of optical silicon using the DOEs method. It was found that 100% similarity between the experimental and acoustic emission prediction and the superiority of cutting speed over both nose radius and rake angle.

The recycling of polymer waste is a way to reduce environmental impacts [31,32,33]. To meet this goal, recycling waste PLA was used to fabricate new PLA filament for fabricating physical models. In addition, the mechanical properties of the physical parts must be sufficient since the mechanical properties of the physical parts depend on the quality of the filament materials greatly. To develop PLA filaments with high tensile strength, an automatic PLA filament extruder was designed and implemented in this study. In general, many researchers used trial-and-error method to investigate process parameters for fabricating PLA filaments. However, the trial-and-error approach will increase production cost and time. The Taguchi coupled with ANOVA is an efficient method in reduction of production cost and time. There is very limited research in the area of recycling PLA polymer to extrude into new filaments. In this study, the DOEs technique is used to investigate the effects of critical process parameters for fabricating PLA filaments with the desired diameter of 1.7 mm. In addition, the confirmation tests were carried out to verify the optimal process parameters obtained by DOEs technique.

## 2. Experiment

**Figure 1:**
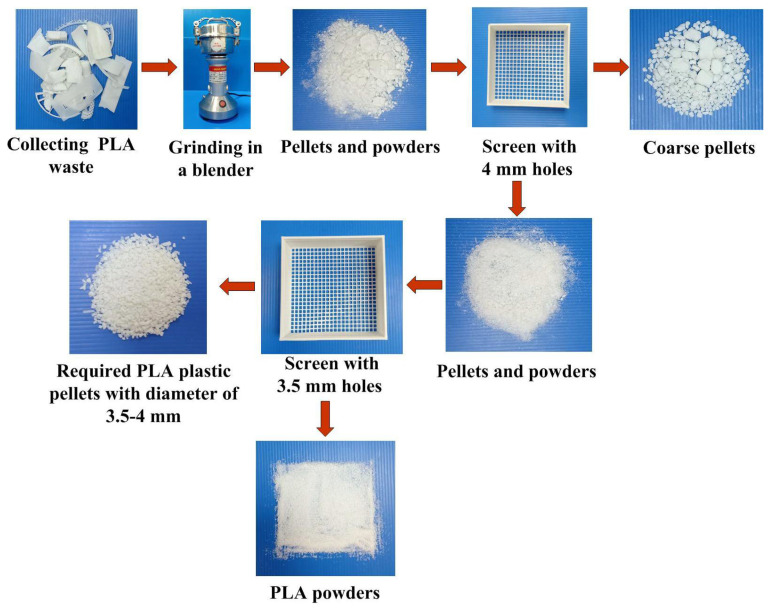
Process workflow for making recycled PLA pellets.

**Figure 2:**
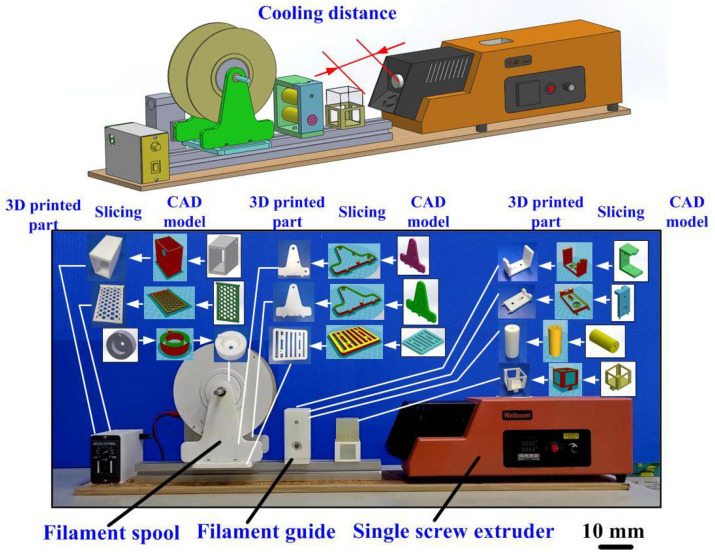
Homemade PLA filament making system.

LA waste plastic is generated through a variety of ways, such and support materials, rafts removed from the physical models, or failed prints. PLA was used to be investigated based on its relative ease of recycling into filament compared to other polymer used for AM. The recycled PLA waste was first recycled for fabricating new PLA filament to fabricate 3D physical models using additive manufacturing technology. To meet sustainability efforts on laboratory to reduce PLA waste plastic, a process workflow process workflow for making recycled PLA pellets was developed in this study, as shown in Figure 1. The manufacturing processes include collecting PLA waste, grinding on a blender (J-150A, Tan Yae Inc., New Taipei City, Taiwan) and screening. Finally, the recycled PLA pallets with average particle size of about 3.5 mm to 4 mm can be obtained. According to the preliminary experiments, four significant factors affecting the PLA filament diameter, i.e. barrel temperature, extrusion speed, cooling distance, and recycled material addition ratio were selected as control factors in this study. Figure 2 shows the homemade PLA filament making system, which was composed of many 3D printed parts built with PLA materials. The relative components for the homemade PLA filament making system were designed using SolidWorks software. 

PLA extracted from corn starch possess some distinct properties, such as low toxicity, repeatability, and high strength. The DOE is useful in process improvement and product development. The features of the DOE include lower development costs, faster time to market as well as lower operating costs. Thus, the Taguchi’s experimental method was used to optimize the process parameters for fabricating PLA filaments with the desired diameter and high tensile strength. To investigate the effects of process parameters on the PLA filament diameter, Taguchi’s L_9_ (3^4^) orthogonal array [34,35,36,37] was employed to determine the signal-to-noise (S/N) ratio in this study. To determine the optimal process parameters for molding tensile test specimens with the desired diameter of 1.7 mm, nominal-the-best [38] was chosen since the FDM 3D printer can print physical models using the PLA filaments smoothly.

## 3. Results and Discussion

**Figure 3:**
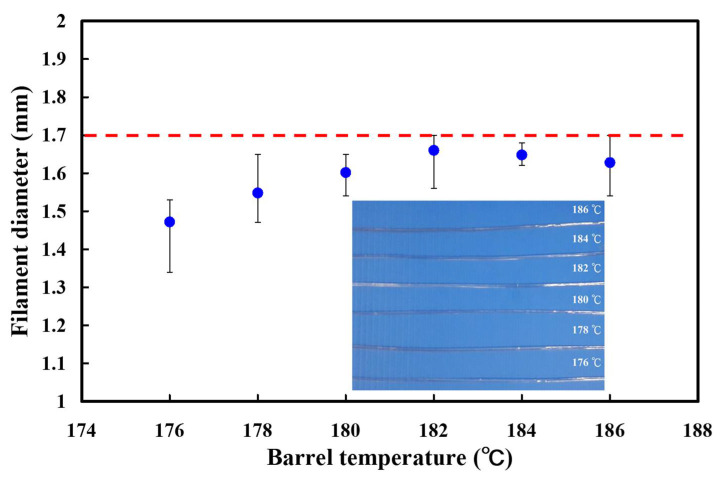
Effects of the barrel temperatures on the PLA filament diameter.

First of all, the one-factor-at-a-time was first used to determine third levels of the four control factors. Figure 3 shows the effects of the barrel temperatures on the PLA filament diameter. A digital caliper was used to measure the diameter of the fabricated filament in five places. The average PLA filament diameters are 1.47 mm, 1.55 mm, 1.60 mm, 1.66 mm, 1.65 mm, and 1.63 mm when the barrel temperatures are 176 °C, 178 °C, 180 °C, 182 °C, 184 °C, and 186 °C, respectively. The standard deviation (SD) of PLA filament diameters are 0.076 mm, 0.075 mm, 0.045 mm, 0.062 mm, 0.027 mm, and 0.067 mm when the barrel temperatures are 176 °C, 178 °C, 180 °C, 182 °C, 184 °C, and 186 °C, respectively. In this study, the desired value of the PLA filament diameter was set at 1.7 mm. In general, the flow rate of material through the nozzle becomes low a when the filament diameter is less than the desired value. In contrast, the filament is not delivered to the nozzle properly when the filament diameter is more than the desired value. The mechanical properties of the fabricated PLA filament were affected greatly by defects, such as voids or air gaps. It should be noted that air gaps or voids inside the PLA filament were found. The average PLA filament diameter is less than desired diameter because the barrel temperature is too low when the barrel temperatures are 176 °C to 180 °C. It should be noted that the roundness of the PLA filament was not acceptable when the barrel temperature is 186 °C because the barrel temperature is too high. The average PLA filament diameter is close to desired diameter when the barrel temperatures are 182 °C and 184 °C. According to the SD of PLA filament diameter, it was found that the PLA filament fabricated by the barrel temperature of 184 °C seems to be the optimal parameter. Thus, the barrel temperature of 184 °C was selected as level 2 of control factor 1. The barrel temperatures of 182 °C and 186 °C were selected as level 1 and level 3 of control factor 1, respectively. 

**Figure 4:**
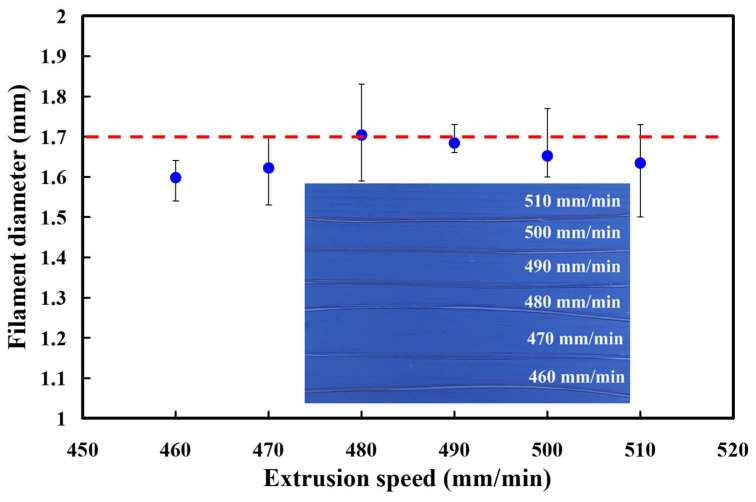
Effects of the extrusion speeds on the PLA filament diameter.

Based on the above results, the barrel temperature was fixed at 184 °C. Six different extrusion speeds were carried out in the following experiment. Figure 4 shows the effects of the extrusion speeds on the PLA filament diameter. The average PLA filament diameters are 1.6 mm, 1.62 mm, 1.7 mm, 1.68 mm, 1.65 mm, and 1.63 mm when the extrusion speeds are 460 mm/min, 470 mm/min, 480 mm/min, 490 mm/min, 500 mm/min, and 510 mm/min, respectively. The SD of PLA filament diameters are 0.043 mm, 0.069 mm, 0.085 mm, 0.027 mm, 0.074 mm, and 0.100 mm when the extrusion speeds are 460 mm/min, 470 mm/min, 480 mm/min, 490 mm/min, 500 mm/min, and 510 mm/min, respectively. As can be seen, the average PLA filament diameter is close to the desired diameter when extrusion speeds are 480 mm/min and 490 mm/min. However, the SD of PLA filament diameter for the extrusion speed of 480 mm/min is larger than that for extrusion speed of 490 mm/min. According to the SD of PLA filament diameter, it was found that the PLA filament fabricated by the extrusion speed of 490 mm/min seems to be the optimal parameter. Thus, the extrusion speed of 490 mm/min was selected as level 2 of control factor 2. The barrel temperatures of 480 mm/min and 500 mm/min were selected as level 1 and level 3 of control factor 2, respectively.

**Figure 5:**
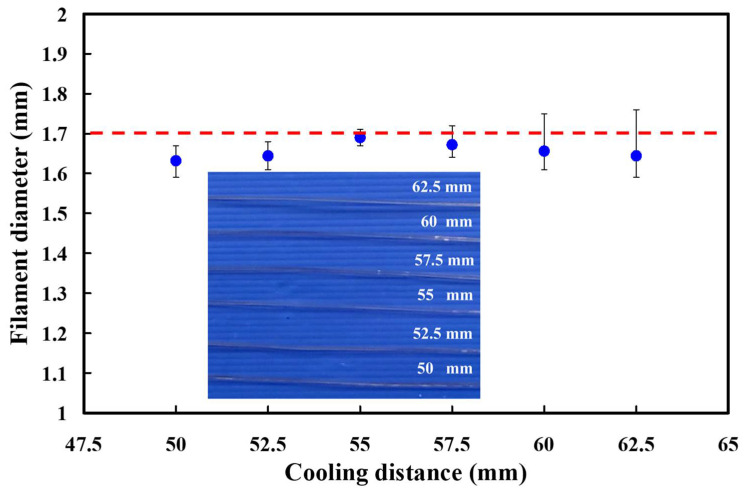
Effects of the cooling distances on the PLA filament diameter.

Figure 5 shows the effects of the cooling distances on the PLA filament diameter. The average PLA filament diameters are 1.63 mm, 1.64 mm, 1.69 mm, 1.67 mm, 1.66 mm, and 1.64 mm when the cooling distances are 50 mm, 52.5 mm, 55 mm, 57.5 mm, 60 mm, and 62.5 mm, respectively. The SD of PLA filament diameters are 0.029 mm, 0.034 mm, 0.016 mm, 0.033mm, 0.055 mm, and 0.069 mm cooling distances are 50 mm, 52.5 mm, 55 mm, 57.5 mm, 60 mm, and 62.5 mm, respectively. As can be seen, the average PLA filament diameter is close to the desired diameter when the cooling distance is 55 mm. It was found that the PLA filament fabricated by the extrusion speed of cooling distance of 55 mm seems to be the optimal parameter. Thus, the cooling distance of 55 mm was selected as level 2 of control factor 3. The cooling distances of 52.5 mm and 57.5 mm were selected as level 1 and level 3 of control factor 3, respectively.

**Figure 6:**
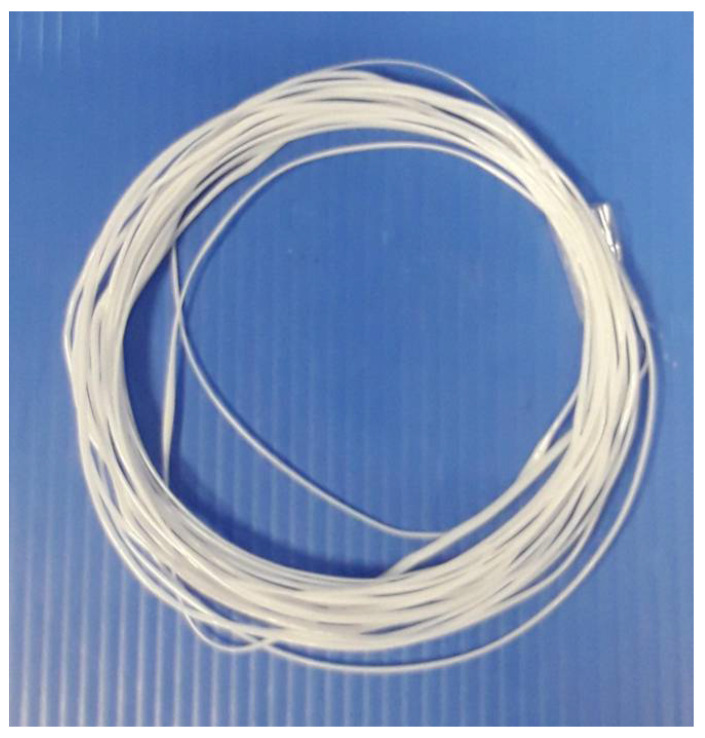
PLA filaments fabricated by recycling material addition ratio of 60%.

**Figure 7:**
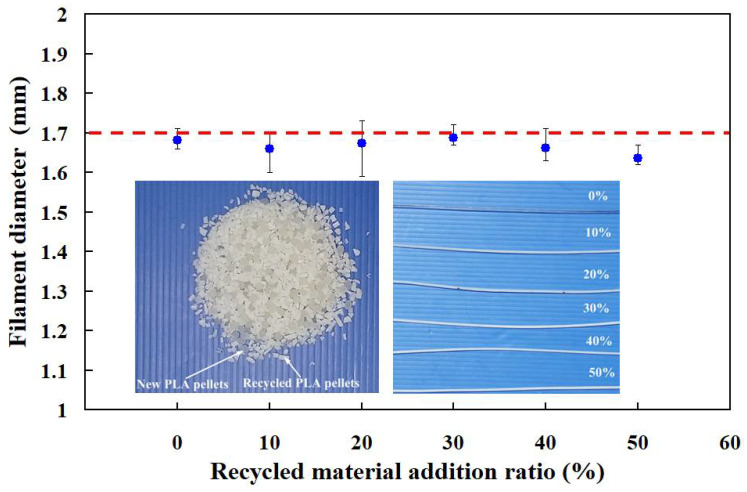
Effects of the recycled material addition ratios on the PLA filament diameter.

The circular economy (CE) is a systemic approach to economic development designed to benefit environment, businesses, and society. To meet the CE, the recycled polylactic acid (PLA) waste was recycled and used to make new PLA filament. Figure 6 shows the PLA filaments fabricated by recycled material addition ratio of 60%. As can be seen, the PLA filament with a uniform diameter can not be produced when the recycling material addition ratio is 60%. The main reason is that the particle size of recycled PLA material is smaller and larger than that of brand new PLA material. As a result, the melting speed of recycled PLA materials is inconsistent with that of brand new PLA materials. Figure 7 shows the PLA filaments fabricated by different recycled material addition ratios. The average PLA filament diameters are 1.68 mm, 1.66 mm, 1.67 mm, 1.69 mm, 1.66 mm, and 1.64 mm when the recycled material addition ratios are 0%, 10%, 20%, 30%, 40%, and 50%, respectively. The standard SD of PLA filament diameters are 0.019 mm, 0.043 mm, 0.057 mm, 0.019 mm, 0.029 mm, and 0.019 mm when the recycled material addition ratios are 0%, 10%, 20%, 30%, 40%, and 50%, respectively. As can be seen, the average PLA filament diameter is close to the desired diameter because the barrel temperature is too low when the recycled material addition ratio is 30%. Thus, the recycled material addition ratio of 30% was selected as level 2 of control factor 4. The recycled material addition ratios of 20% and 40% were selected as level 1 and level 3 of control factor 4, respectively. According to the preliminary experimental results described above, four process control factors and their levels were summarized in the Table 1.

**Figure 8:**
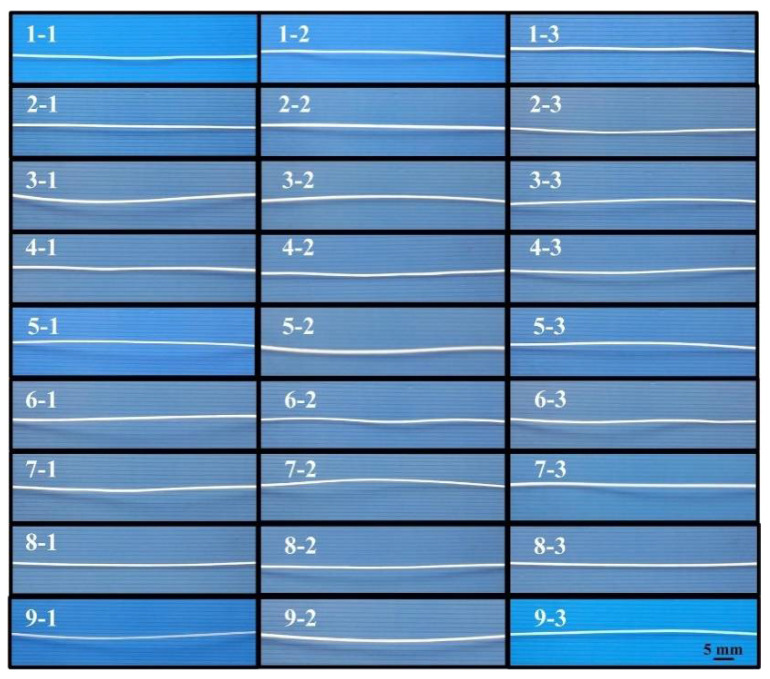
Results of PLA filament fabricated by different process parameters.

In general, the Taguchi method [39] has three quality characteristics, i.e. the-bigger-the-better, the-nominal-the-best, and the-smaller-the-better. The orthogonal array (OA) [40] was used in this study because it is suitable for the four process control factors with three levels. To investigate the optimal process parameters for manufacturing PLA filaments with the desired value of diameter, three levels were employed in this study. The experimental results of the average diameter of PLA filament based on L_9_ OA were listed in the Table 2. Figure 8 shows the results of PLA filament fabricated by different process parameters. In this study, the-nominal-the-better was used because the desired value of filament diameter is 1.7 mm which is suitable for printing physical models. The corresponding S/N ratio can be calculated based on the-nominal-the-better quality characteristics, as shown in Table 3.

**Figure 9:**
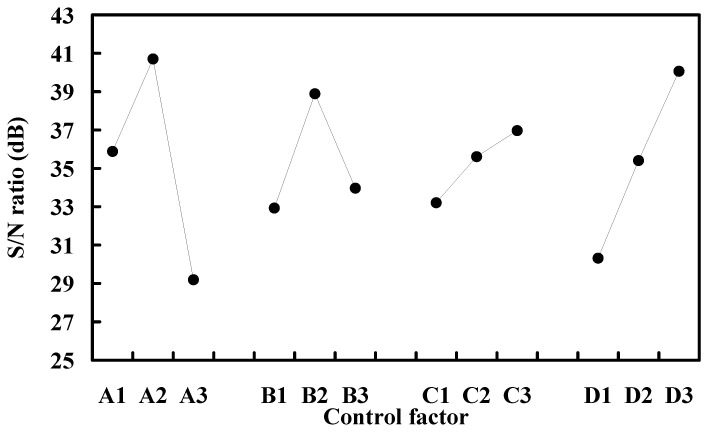
S/N ratio effects of each process control factor.

The S/N ratio effects of each process control factor are shown in the Figure 9. As can be seen, a set of optimal combination of process control factors and levels can be determined directly according to the higher S/N ratio. The optimal combination is A2, B2, C3, and D3. The optimal process parameters for manufacturing the with the desired diameter include the barrel temperature of 184 °C, extrusion speed of 490 mm/min, cooling distance of 57.5 mm, and recycled material addition ratio of 40%.

**Figure 10:**
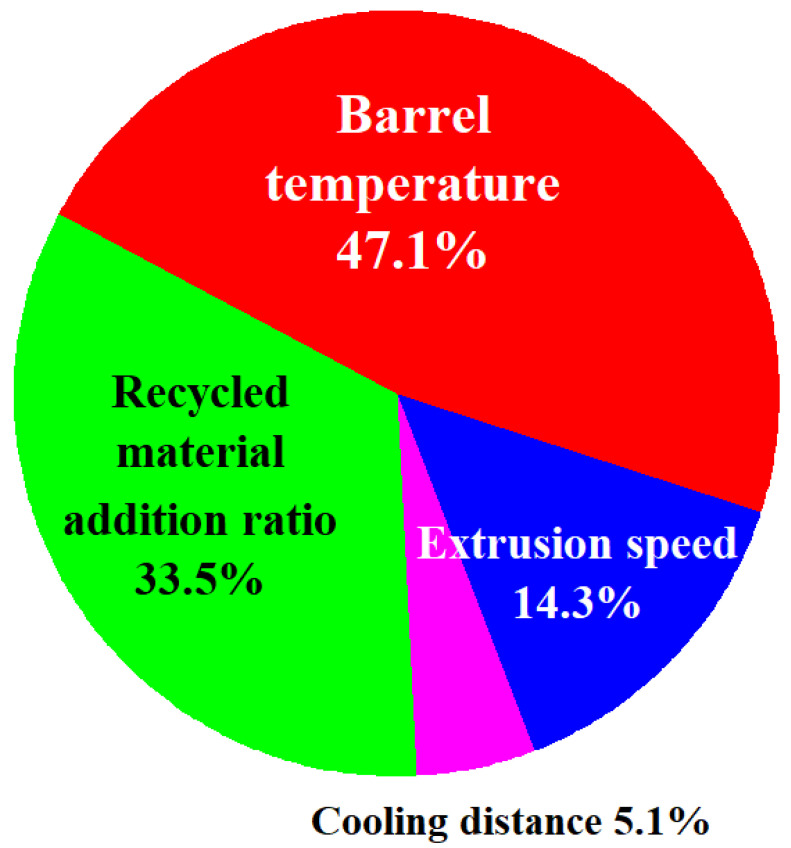
Percentage contributions of the four different control factors

The ANOVA coupled with Taguchi method was performed in order to investigate the results of the Taguchi method. The percentage contribution of each control factor was employed to evaluate the corresponding effect on the quality characteristic. The results of ANOVA were listed in the Table 4. The percentage contribution of each control factor can be calculated. As can be seen, the percentage contributions of the four different control factors a, b, c, and d are 47.1%, 14.3%, 5.1%, and 33.5%, respectively. It is interesting to note that the barrel temperature is the most significant control factor affecting the diameter of PLA filament followed by recycling material addition ratio, extrusion speed, and cooling distance. This result was consistent with the observed results during the experiment since the fluidity of the molten material was affected by the barrel temperature significantly. Figure 10 shows the percentage contributions of the four different control factors.

**Figure 11:**
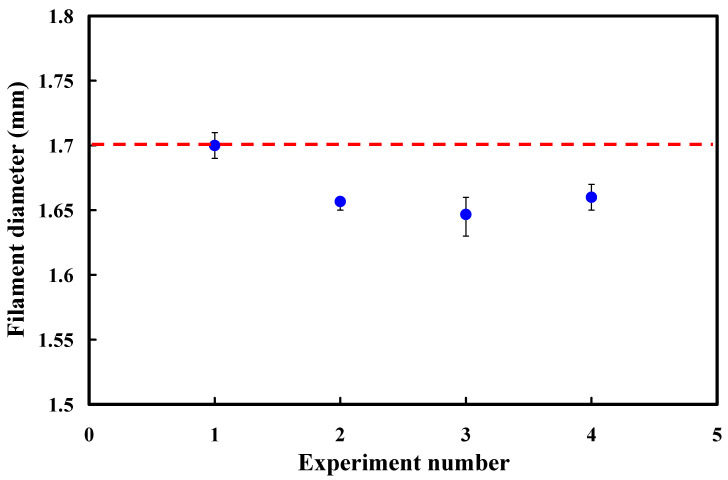
Results of the verification experiments.

The verification experiment is essential in engineering analysis to validate the diameter of the PLA filament fabricated by the optimal process parameters. Thus, the final step of the DOEs is to carry out a verification experiment for examining diameter of the PLA filaments fabricated by both the general process parameters and optimal process parameters. To verify the effectiveness of the optimal process parameters, three sets of general process parameters were employed to manufacture PLA filaments. Table 5 shows the results of verifying the optimal process parameters. Figure 11 shows the results of the verification experiments. The PLA filament diameters fabricated by the optimal process parameters (No. 1 of verification experiments) are 1.7 mm, 1.71 mm, and 1.69 mm, respectively. The average PLA filament diameter is approximately 1.7 mm. The PLA filament diameters fabricated by the first general process parameters (No. 2 of verification experiments) are 1.66 mm, 1.65 mm, and 1.66 mm, respectively. The average PLA filament diameter is approximately 1.66 mm. The PLA filament diameters fabricated by the second general process parameters (No. 3 of verification experiments) are 1.63 mm, 1.65 mm, and 1.66 mm, respectively. The average PLA filament diameter is approximately 1.65 mm. The PLA filament diameters fabricated by the third general process parameters (No. 4 of verification experiments) are 1.62 mm, 1.61 mm, and 1.65 mm, respectively. The average PLA filament diameter is approximately 1.63 mm. It should be noted that the results were in good agreement with results from ANOVA optimal level. This shows that the average diameter of the PLA filament fabricated by the optimal process parameters is obviously better than that of the PLA filament fabricated by the general process parameters.

**Figure 12:**
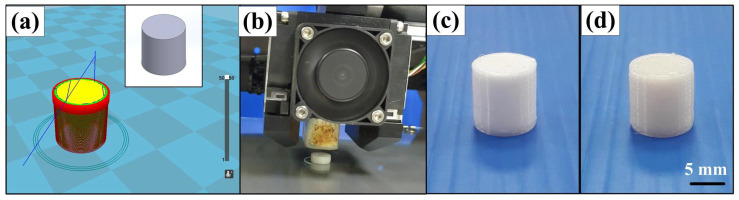
Effectiveness evaluation process (**a**) CAD model of the cylinder and slicing results, (**b**) printing situation of the cylindrical prototype, (**c**) a typical cylindrical prototype built with commercial PLA filaments, and (**d**) a typical cylindrical prototype built with commercially available PLA filament developed in this study.

To evaluate the performance of the PLA filament fabricated, a cylindrical part with a diameter and height of 10 mm and 10 mm was designed for printing using both commercially available PLA filament and PLA filament developed in this study. The process parameters for printing cylinder prototype include a printing temperature of 210 °C, hot bed temperature of 70 °C, printing speed of 20 mm/s, and filling density of 30%. To verify the effectiveness of the PLA filament fabricated in this study, thirty parts were printed and evaluated. Figure 12 shows the effectiveness evaluation process. This means recycling PLA waste plastic is a viable option since the cylindrical prototypes can be built layer by layer via the extruded PLA filaments. The diameter and height of the printed cylinder prototype built with commercially available PLA filament are 10.16 mm and 10.26 mm, respectively. The diameter and height of the printed cylinder prototype built with PLA filament developed in this study are 10.18 mm and 10.22 mm, respectively. This means that the diameter and height of the printed cylindrical prototype built with PLA filament developed in this study are very close to those built with commercially available PLA filament. The only difference is that the appearance of the cylindrical prototype built with the PLA filament developed in this study is lighter since the filament developed in this study contains 40% recycled PLA material.

**Figure 13:**
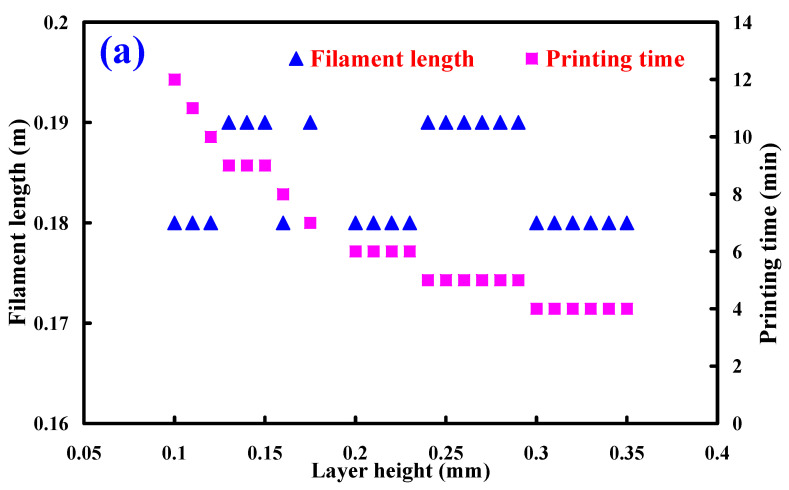

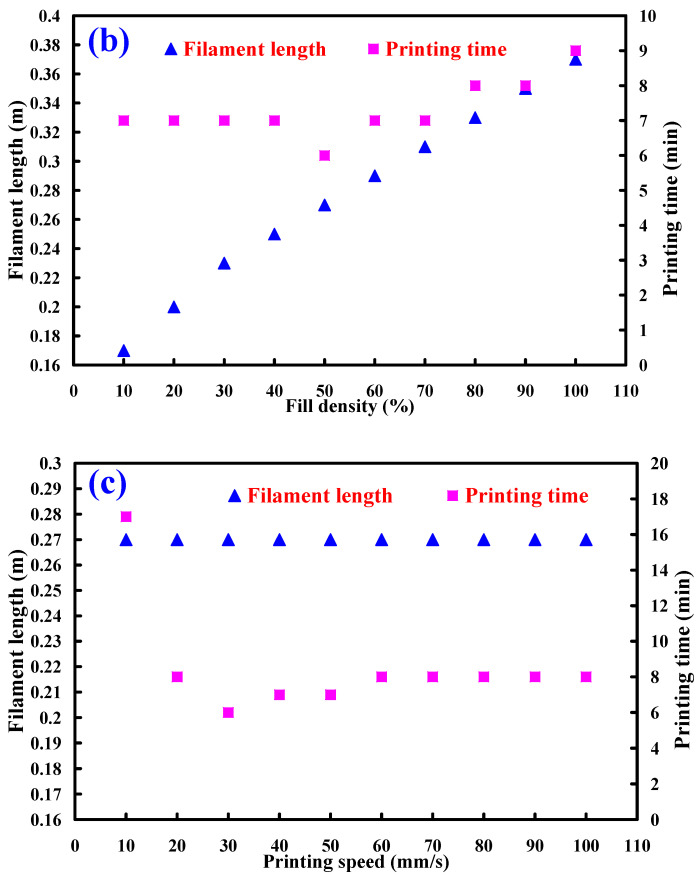
Filament length and printing time as a function of different (**a**) layer heights, (**b**) fill densities, and (**c**) printing speeds.

Figure 13 shows the filament length and printing time as a function of different layer heights, fill densities, and printing speeds. The surface quality of the cylinder prototypes is affected by the layer height greatly. Generally, the printing time is shorter when the layer thickness is thicker. However, cylinder prototype has poor surface quality because of the staircase effect. In contrast, the printing time is longer when the layer thickness is thinner. To improve the surface quality of the prototype, several methods have been proposed including chemical finishing, analytical models, adaptive slicing, and hot cutter machining. Table 6 shows the process parameters for making physical prototype. Based on the results of a series of experiments, it can be used as the standard process parameters for rapid tooling application.

**Figure 14:**
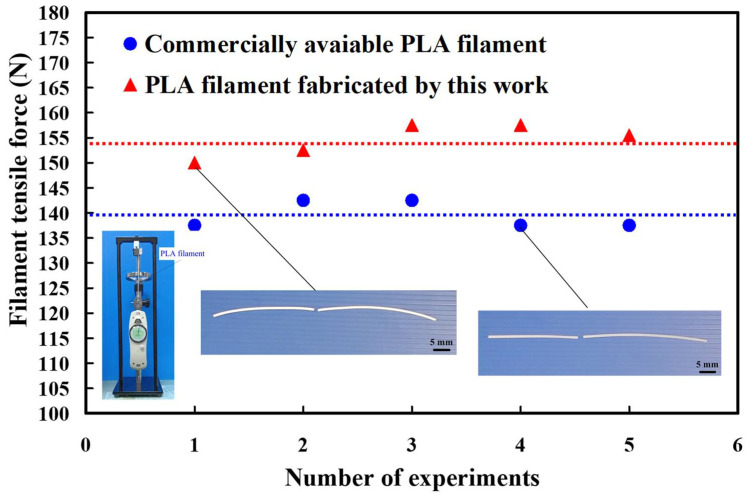
Filament tensile force of the two kinds of PLA filaments.

The fused filament fabrication (FFF) [41] is a form of polymer additive manufacturing (AM). Commercially available PLA filaments are widely used for FFF. To verify the difference in filament tensile force between the PLA filaments produced by this work and the commercially available PLA filament. Figure 14 shows the filamenr tensile force of the two kinds of PLA filaments. The average tensile force of the commercially available PLA filaments is about respectively 139.5 N. However, the average tensile force of the PLA filaments produced by this work is about respectively 154.6 N. These results indicate that the tensile force of the PLA filament produced by this work is about 1.1 times that of the commercially available PLA filament. This means that the developed PLA filaments can be used for direct soft tooling [42,43] application since the mold fabricated possesses high tensile strength [44,45], which can be used for small-volumn production. The commercially available PLA material is NTD 0.25/g. The cost of producing a five-meter-long filament using commercially available PLA material is about NTD 3.53 since it is necessary to use 14.1 g of commercially available PLA material. However, the cost of producing a five-meter-long filament using commercially available PLA material is about NTD 2.12 since it is necessary to use 8.46 g of commercially available PLA material and 5.64 g recycled PLA materials. This means that about 40% savings in production costs can be obtained for producing a five-meter-long filament using the PLA filament produced by this work. According to the above findings, the developed PLA filaments are very practical and provide the greatest application potential in the industry because the PLA filaments can be used to fabricate three-dimensional models with high tensile strength under the condition of low manufacturing cost. In this study, the PLA waste was used to fabricate new filament. A wide range of materials, including high-density polyethylene (HDPE) [46], PA, ABS, Nylon [47], polypropylene (PP) [48], and glass-filled nylon [49] can also be recycled to manufacturing new filament. Metallic powders, such as copper powder [50] or aluminum powder [51] can also be added in the manufacturing process to improve the physical properties of the fabricated filament. In addition, some reinforcing fillers, such as silicon carbide, aluminum oxide, molybdenum disulfide [52,53,54], zirconia [55,56,57], or silicon nitride [58,59,60] can also be mixed into the recycled PLA materials to develop filaments with high wear resistance [61] to enhance the life of direct soft tooling built with filaments [62,63,64].Therefore, the filament with both excellent mechanical and physical properties can be employed to fabricate rapid tooling [65] for thermoforming, blow molding [66], plastic injection molding [67,68], rotational molding, or transfer molding. These issues are currently being investigated and the results will be presented in a later study. 

## 4. Conclusions

The amount of PLA waste plastic is increasing due to the annual growth rate of 3D polymer printing. To reduce the amount of PLA waste plastic for green manufacturing, the recycled PLA waste plastic was used to manufacture new PLA for printing physical models using AM technology. In this study, the Taguchi method was used to investigate the optimal levels of process conditions for fabricating PLA filaments with the desired value of diameter. The main conclusions from the experimental work in this study are as follows:

1. The developed PLA filaments are very practical and provide the greatest application potential in the AM industry since the PLA filaments can be employed to print physical models with high tensile strength economically.
2. The tensile strength of the developed PLA filament is approximately 1.1 times that of the commercially available PLA filament. The production cost is only 60% of the commercially available PLA filament.
3. The most important control factor affecting the diameter of the PLA filament is the barrel temperature, followed by the recycling material addition ratio, extrusion speed, and cooling distance.
4. The optimal process parameters for fabricating PLA filaments with a diameter of 1.7 mm include a barrel temperature of 184 °C, extrusion speed of 490 mm/min, cooling distance of 57.5 mm, and recycled material addition ratio of 40%.

## Figures and Tables

**Table 1:** Process control factors and their levels.

Control Factor		Level 1	Level 2	Level 3
A	Barrel temperature (°C)	182	184	186
B	Extrusion speed (mm/min)	480	490	500
C	Cooling distance (mm)	52.5	55	57.5
D	Recycled material addition ratio (%)	20	30	40

**Table 2:** Experimental results of average diameter of PLA filament.

Experiment No.	Control Factor				Average Diameter (mm)			σ^2^	S/N (dB)
	A	B	C	D	1	2	3		
1	A1	B 1	C 1	D 1	1.66	1.65	1.65	0.0058	26.55
2	A1	B 2	C 2	D 2	1.7	1.69	1.71	0.0100	40.00
3	A1	B 3	C 3	D 3	1.7	1.69	1.69	0.0058	41.09
4	A2	B 1	C 2	D 3	1.7	1.7	1.69	0.0058	43.52
5	A2	B 2	C 3	D 1	1.69	1.69	1.7	0.0058	41.09
6	A 2	B 3	C 1	D 2	1.68	1.7	1.7	0.0115	37.50
7	A3	B 1	C 3	D 2	1.65	1.68	1.67	0.0153	28.71
8	A3	B 2	C 1	D 3	1.69	1.68	1.71	0.0153	35.56
9	A3	B 3	C 2	D 1	1.63	1.62	1.65	0.0153	23.30

**Table 3:** Response table of S/N ratio based on the-larger-the-better quality characteristics.

Control Factor	Level 1	Level 2	Level 3
Barrel temperature (°C)	35.8818	40.7048	29.1924
Extrusion speed (mm/min)	32.9301	38.8848	33.9641
Cooling distance (mm)	33.2060	35.6071	36.9658
Recycled material addition ratio (%)	30.3150	35.4053	40.0588

**Table 4:** ANOVA table.

Control Factor		Leve1	Level 2	Level 3	SS	DOF	V	ρ (%)
A	Barrel temperature (°C)	35.8818	40.7048	29.1924	200.545	2	100.272	47.1
B	Extrusion speed (mm/min)	32.9301	38.8848	33.9641	60.741	2	30.370	14.3
C	Cooling distance (mm)	33.2060	35.6071	36.9658	21.747	2	10.873	5.1
D	Recycled material addition ratio (%)	30.3150	35.4053	40.0588	142.507	2	71.253	33.5

**Table 5:** Results of verifying the optimal process parameters.

No. of Verification Experiments	Process Parameters	Diameter of PLA Filament (mm)			
		1	2	3	Average
1	Barrel temperature 184 °C Extrusion speed 500 mm/min Cooling distance 57.5 mm Recycled material addition ratio 30%	1.7	1.69	1.69	1.69
2	Barrel temperature 186 °C Extrusion speed 480 mm/min Cooling distance 52.5 mm Recycled material addition ratio 20%	1.66	1.65	1.66	1.66
3	Barrel temperature 184 °C Extrusion speed 480 mm/min Cooling distance 57.5 mm Recycled material addition ratio 30%	1.63	1.65	1.66	1.65
4	Barrel temperature 182 °C Extrusion speed 500 mm/min Cooling distance 55 mm Recycled material addition ratio 40%	1.62	1.61	1.65	1.63

**Table 6:** Process parameters for making physical prototype.

Parameter	Input Values
Layer height (mm)	0.18
Shell thickness (mm)	0.7
Bottom/top thickness (mm)	0.6
Fill density (%)	40–50
Print speed (mm/s)	30–40
Printing temperature (°C)	220
Heated bed (°C)	0–50
Platform adhesion type	Raft
Filament diameter (mm)	1.75
Flow (%)	100
Retraction speed (mm/s)	45
Retraction distance (mm)	3
Initial layer thickness (mm)	0.26
Initial layer line width (%)	100
Dual extrusion overlap (mm)	0.15
Travel speed (mm/s)	100
Bottom layer speed (mm/s)	20
Minimal layer thickness (s)	5
Enable cooling fan	on

## Data Availability

Data sharing is available on request from corresponding author.

## References

[1] Rehmani M.A.A., Jaywant S.A., Arif K.M. (2021). Study of Microchannels Fabricated Using Desktop Fused Deposition Modeling Systems. Micromachines.

[2] Pitaru A.A., Lacombe J.-G., Cooke M.E., Beckman L., Steffen T., Weber M.H., Martineau P.A., Rosenzweig D.H. (2020). Investigating Commercial Filaments for 3D Printing of Stiff and Elastic Constructs with Ligament-Like Mechanics. Micromachines.

[3] Kotz F., Mader M., Dellen N., Risch P., Kick A., Helmer D., Rapp B.E. (2020). Fused Deposition Modeling of Microfluidic Chips in Polymethylmethacrylate. Micromachines.

[4] Xu S., Huang J., Liu J., Ma Y. (2020). Topology Optimization for FDM Parts Considering the Hybrid Deposition Path Pattern. Micromachines.

[5] Liu Z., Wang Y., Wu B., Cui C., Guo Y., Yan C. (2019). A critical review of fused deposition modeling 3D printing technology in manufacturing polylactic acid parts. Int. J. Adv. Manuf. Technol..

[6] Adesina O.T., Sadiku E.R., Jamiru T., Ogunbiyi O.F., Beneke L.W., Adegbola A.T. (2019). Optimization of SPS processing parameters on the density and hardness properties of graphene reinforced polylactic acid nanocomposite. Int. J. Adv. Manuf. Technol..

[7] Yu W., Ruan S., Li Z., Gu J., Wang X., Shen C., Chen B. (2019). Effect of injection velocity on the filling behaviors of microinjection-molded polylactic acid micropillar array product. Int. J. Adv. Manuf. Technol..

[8] Daniel F., Patoary N.H., Moore A.L., Weiss L., Radadia A.D. (2018). Temperature-dependent electrical resistance of conductive polylactic acid filament for fused deposition modeling. Int. J. Adv. Manuf. Technol..

[9] Li H., Wang T., Li Q., Yu Z., Wang N. (2018). A quantitative investigation of distortion of polylactic acid/PLA) part in FDM from the point of interface residual stress. Int. J. Adv. Manuf. Technol..

[10] Lebedev S.M. (2019). Manufacturing poly(lactic acid)/metal composites and their characterization. Int. J. Adv. Manuf. Technol..

[11] Mohamed O.A., Masood S.H., Bhowmik J.L. (2015). Optimization of fused deposition modeling process parameters: A review of current research and future prospects. Adv. Manuf..

[12] Lee W.C., Wei C.C., Chung S.C. (2014). Development of a hybrid rapid prototyping system using low-cost fused deposition modeling and five-axis machining. J. Mater. Process. Technol..

[13] Drummer D., Cifuentes-Cuéllar S., Rietzel D. (2012). Suitability of PLA/TCP for fused deposition modeling. Rapid Prototyp. J..

[14] Perez A.R.T., Roberson D.A., Wicker R.B. (2014). Fracture Surface Analysis of 3D-Printed Tensile Specimens of Novel ABS-Based Materials. J. Fail. Anal. Prev..

[15] Berrio Bernal J.D., Silva E.C.N., Montealegre Rubio W. (2019). Characterization of effective Young’s modulus for Fused Deposition Modeling manufactured topology optimization designs. Int. J. Adv. Manuf. Technol..

[16] Lin S., Xia L., Ma G., Zhou S., Xie Y.M. (2019). A maze-like path generation scheme for fused deposition modeling. Int. J. Adv. Manuf. Technol..

[17] Zekavat A.R., Jansson A., Larsson J., Pejryd L. (2019). Investigating the effect of fabrication temperature on mechanical properties of fused deposition modeling parts using X-ray computed tomography. Int. J. Adv. Manuf. Technol..

[18] Baca D., Ahmad R. (2020). The impact on the mechanical properties of multi-material polymers fabricated with a single mixing nozzle and multi-nozzle systems via fused deposition modeling. Int. J. Adv. Manuf. Technol..

[19] Paggi R.A., Salmoria G.V., Ghizoni G.B., Back H.D.M., Gindri I.D.M. (2019). Structure and mechanical properties of 3D-printed cellulose tablets by fused deposition modeling. Int. J. Adv. Manuf. Technol..

[20] Camposeco-Negrete C. (2020). Optimization of printing parameters in fused deposition modeling for improving part quality and process sustainability. Int. J. Adv. Manuf. Technol..

[21] Liu W., Li Y., Liu B., Wang G. (2020). Development of a novel rectangular–circular grid filling pattern of fused deposition modeling in cellular lattice structures. Int. J. Adv. Manuf. Technol..

[22] Zaman U.K.U., Boesch E., Siadat A., Rivette M., Baqai A.A. (2019). Impact of fused deposition modeling (FDM) process parameters on strength of built parts using Taguchi’s design of experiments. Int. J. Adv. Manuf. Technol..

[23] Adnan M.F., AbdullahE A.B., Samad Z. (2017). Springback behavior of AA6061 with non-uniform thickness section using Taguchi Method. Int. J. Adv. Manuf. Technol..

[24] Zhou M., Kong L., Xie L., Fu T., Jiang G., Feng Q. (2017). Design and optimization of non-circular mortar nozzles using finite volume method and Taguchi method. Int. J. Adv. Manuf. Technol..

[25] Azadeh A., Gharibdousti M.S., Firoozi M., Baseri M., Alishahi M., Salehi V. (2016). Selection of optimum maintenance policy using an integrated multi-criteria Taguchi modeling approach by considering resilience engineering. Int. J. Adv. Manuf. Technol..

[26] Effertz P.S., Quintino L., Infante V. (2017). The optimization of process parameters for friction spot welded 7050-T76 aluminium alloy using a Taguchi orthogonal array. Int. J. Adv. Manuf. Technol..

[27] Feng Q., Liu L., Zhou X. (2020). Automated multi-objective optimization for thin-walled plastic products using Taguchi, ANOVA, and hybrid ANN-MOGA. Int. J. Adv. Manuf. Technol..

[28] Cherief M., Belaadi A., Bouakba M., Bourchak M., Meddour I. (2020). Behaviour of lignocellulosic fibre-reinforced cellular core under low-velocity impact loading: Taguchi method. Int. J. Adv. Manuf. Technol..

[29] Oemar B., Chang W. (2020). Taguchi method for optimizing process parameters in the production of activated carbon from rubber seed shell. Int. J. Adv. Manuf. Technol..

[30] Abdulkadir L.N., Abou-El-Hossein K., Abioye A.M., Liman M.M., Cheng Y.-C., Abbas A.A.S. (2019). Process parameter selection for optical silicon considering both experimental and AE results using Taguchi L9 orthogonal design. Int. J. Adv. Manuf. Technol..

[31] Li K., Zhou T., Liu B. (2020). Internet-based intelligent and sustainable manufacturing: Developments and challenges. Int. J. Adv. Manuf. Technol..

[32] Yu H., Lyu Y., Wang J. (2019). Green manufacturing with a bionic surface structured grinding wheel-specific energy analysis. Int. J. Adv. Manuf. Technol..

[33] Wang X., Chen L., Dan B., Wang F. (2018). Evaluation of EDM process for green manufacturing. Int. J. Adv. Manuf. Technol..

[34] Camposeco-Negrete C. (2019). Prediction and optimization of machining time and surface roughness of AISI O1 tool steel in wire-cut EDM using robust design and desirability approach. Int. J. Adv. Manuf. Technol..

[35] Hirigo T.H., Singh B. (2019). Design and analysis of sand casting process of mill roller. Int. J. Adv. Manuf. Technol..

[36] Revuru R.S., Zhang J.Z., Posinasetti N.R. (2020). Comparative performance studies of turning 4140 steel with TiC/TiCN/TiN-coated carbide inserts using MQL, flooding with vegetable cutting fluids, and dry machining. Int. J. Adv. Manuf. Technol..

[37] Tzeng S., Chen X., Wang W. (2020). Numerical studies of metal particle behaviors inside the selective laser melting (SLM) chamber through computational fluid dynamics (CFD). Int. J. Adv. Manuf. Technol..

[38] Wu F., Chyu C. (2002). A Comparative Study on Taguchi’s SN Ratio, Minimising MSD and Variance for Nominal-the-Best Characteristic Experiment. Int. J. Adv. Manuf. Technol..

[39] Hentati F., Hadriche I., Masmoudi N., Bradai C. (2019). Optimization of the injection molding process for the PC/ABS parts by integrating Taguchi approach and CAE simulation. Int. J. Adv. Manuf. Technol..

[40] Ujah C.O., Popoola A.P.I., Popoola O.M., Aigbodion V.S. (2019). Optimisation of spark plasma sintering parameters of Al-CNTs-Nb nano-composite using Taguchi Design of Experiment. Int. J. Adv. Manuf. Technol..

[41] Vidakis N., Petousis M., Maniadi A., Koudoumas E., Kenanakis G., Romanitan C., Tutunaru O., Suchea M., Kechagias J. (2020). The Mechanical and Physical Properties of 3D-Printed Materials Composed of ABS-ZnO Nanocomposites and ABS-ZnO Microcomposites. Micromachines.

[42] Dempsey D., McDonald S., Masato D., Barry C. (2020). Characterization of Stereolithography Printed Soft Tooling for Micro Injection Molding. Micromachines.

[43] Martínez-López J.I., Betancourt Cervantes H.A., Cuevas Iturbe L.D., Vázquez E., Naula E.A., Martínez López A., Siller H.R., Mendoza-Buenrostro C., Rodríguez C.A. (2020). Characterization of Soft Tooling Photopolymers and Processes for Micromixing Devices with Variable Cross-Section. Micromachines.

[44] Rajpurohit S.R., Dave H.K. (2019). Analysis of tensile strength of a fused filament fabricated PLA part using an open-source 3D printer. Int. J. Adv. Manuf. Technol..

[45] Kuo C., Liu H., Chang C. (2020). Optimization of vacuum casting process parameters to enhance tensile strength of components using design of experiments approach. Int. J. Adv. Manuf. Technol..

[46] Vital-Grappin A.D., Ariza-Tarazona M.C., Luna-Hernández V.M., Villarreal-Chiu J.F., Hernández-López J.M., Siligardi C., Cedillo-González E.I. (2021). The Role of the Reactive Species Involved in the Photocatalytic Degradation of HDPE Microplastics Using C,N-TiO_2_ Powders. Polymers.

[47] Tong X., Wang Z., Zhang M.-L., Wang X.-J., Zhang G., Long S.-R., Yang J. (2021). Synthesis, Characterization and Non-Isothermal Crystallization Kinetics of a New Family of Poly (Ether-Block-Amide)s Based on Nylon 10T/10I. Polymers.

[48] Pisanu L., Santiago L.C., Barbosa J.D.V., Beal V.E., Nascimento M.L.F. (2021). Effect of the Process Parameters on the Adhesive Strength of Dissimilar Polymers Obtained by Multicomponent Injection Molding. Polymers.

[49] Sanya O.T., Oji B., Owoeye S.S., Egbochie E.J. (2019). Influence of particle size and particle loading on mechanical properties of silicon carbide–reinforced epoxy composites. Int. J. Adv. Manuf. Technol..

[50] Abima C.S., Akinlabi E.T., Akinlabi S.A., Fatoba O.S., Oladijo O.P. (2019). Microstructural, mechanical and corrosion properties of aluminium MIG welds reinforced with copper powder. Int. J. Adv. Manuf. Technol..

[51] Manap A., Okabe T., Ogawa K., Mahalingam S., Abdullah H. (2019). Experimental and smoothed particle hydrodynamics analysis of interfacial bonding between aluminum powder particles and aluminum substrate by cold spray technique. Int. J. Adv. Manuf. Technol..

[52] Azarhoushang B., Soltani B., Zahedi A. (2017). Laser-assisted grinding of silicon nitride by picosecond laser. Int. J. Adv. Manuf. Technol..

[53] Laouissi A., Yallese M.A., Belbah A., Belhadi S., Haddad A. (2019). Investigation, modeling, and optimization of cutting parameters in turning of gray cast iron using coated and uncoated silicon nitride ceramic tools. Based on ANN, RSM, and GA optimization. Int. J. Adv. Manuf. Technol..

[54] Kim T.W., Lee C.M. (2015). A study on the development of milling process for silicon nitride using ball end-mill tools by laser-assisted machining. Int. J. Adv. Manuf. Technol..

[55] Yang M., Li C., Zhang Y., Jia D., Li R., Hou Y., Cao H. (2019). Effect of friction coefficient on chip thickness models in ductile-regime grinding of zirconia ceramics. Int. J. Adv. Manuf. Technol..

[56] Xu S., Yao Z., Cai H., Wang H. (2017). An experimental investigation of grinding force and energy in laser thermal shock-assisted grinding of zirconia ceramics. Int. J. Adv. Manuf. Technol..

[57] Abdo B.M.A., El-Tamimi A.M., Anwar S., Umer U., Alahmari A.M., Ghaleb M.A. (2018). Experimental investigation and multi-objective optimization of Nd:YAG laser micro-channeling process of zirconia dental ceramic. Int. J. Adv. Manuf. Technol..

[58] Yao J., Wu Y., Sun J., Ying X., He W., Peng Z. (2020). Research on the metamorphic layer of silicon nitride ceramic under high temperature based on molecular dynamics. Int. J. Adv. Manuf. Technol..

[59] Zhenhua W., Ning S., Liyan C., Zengbin Y., Yulin W., Juntang Y. (2020). Cutting performance and wear mechanism of spark plasma–sintered silicon nitride ceramics tool in dry turning of 41Cr4 hardened steel. Int. J. Adv. Manuf. Technol..

[60] Minghua C., Wenqi Z., Jiawei Z., Oingguo C. (2020). Dielectric Property and Space Charge Behavior of Polyimide/Silicon Nitride Nanocomposite Films. Polymers.

[61] Li A., Lan Q., Dong D., Liu Z., Li Z., Bian Y. (2014). Integrated design and process analysis of a blow molding turbo-charged pipe. Int. J. Adv. Manuf. Technol..

[62] Fu J., Ma Y. (2019). A method to predict early-ejected plastic part air-cooling behavior towards quality mold design and less molding cycle time. Robot. Comput. Integr. Manuf..

[63] Kitayama S., Yokoyama M., Takano M., Aiba S. (2017). Multi-objective optimization of variable packing pressure profile and process parameters in plastic injection molding for minimizing warpage and cycle time. Int. J. Adv. Manuf. Technol..

[64] Krebelj K., Halilovič M., Mole N. (2019). The cooling rate dependence of the specific volume in amorphous plastic injection molding. Int. J. Adv. Manuf. Technol..

[65] Hsieh Y.C., Doan M.H. (2018). Research on both the radiation heating and the cooling system inside the stretch blow molding machine CPSB-LSS12. Int. J. Adv. Manuf. Technol..

[66] Kuo C.-C., Nguyen T.-D., Zhu Y.-J., Lin S.-X. (2021). Rapid Development of an Injection Mold with High Cooling Performance Using Molding Simulation and Rapid Tooling Technology. Micromachines.

[67] Thanh T.D., Tran M.T.U., Pham S.M. (2021). The Feasibility of an Internal Gas-Assisted Heating Method for Improving the Melt Filling Ability of Polyamide 6 Thermoplastic Composites in a Thin Wall Injection Molding Process. Polymers.

[68] Kitayama S., Hashimoto S., Takano M., Yamazaki Y., Kubo Y., Aiba S. (2020). Multi-objective optimization for minimizing weldline and cycle time using variable injection velocity and variable pressure profile in plastic injection molding. Int. J. Adv. Manuf. Technol..

